# Hepatic FGF21: Its Emerging Role in Inter-Organ Crosstalk and Cancers

**DOI:** 10.7150/ijbs.76924

**Published:** 2022-10-03

**Authors:** Yue SUI, Jianping CHEN

**Affiliations:** 1 School of Chinese Medicine, The University of Hong Kong, Pokfulam, Hong Kong, China.; 2 Shenzhen Institute of Research and Innovation, The University of Hong Kong, Shenzhen, China.

**Keywords:** Fibroblast growth factor 21, Hepatic FGF21, Endocrine FGF21, Liver, Inter-organ crosstalk, Cancer

## Abstract

Fibroblast growth factor (FGF) 21 is one of the FGF members with special endocrine properties. In the last twenty years, it has attracted intense research and development for its physiological functions that respond to dietary manipulation, pharmacological benefits of improving the macronutrient metabolism, and clinical values as a biomarker of various human diseases. Generally, FGF21 can be produced by major metabolic organs, but only the subgroup from the liver shows canonical endocrine properties, which emphasizes the special value of delineating the unique secretory and functional characteristics of hepatic FGF21. There has been a growth in literature to address the extra-hepatic activities of FGF21, and many striking findings have therefore been published. Yet, they are fragmented and scattered, and controversies are raised from divergent findings. For this reason, there is a need for a systematic and critical evaluation of current research in this aspect. In this review, we focus on the current knowledge about the molecular biology of endocrine FGF21, especially present details on the regulation of circulating levels of FGF21. We also emphasize its emerging roles in inter-organ crosstalk and cancer development.

## Introduction

**Figure 1:**
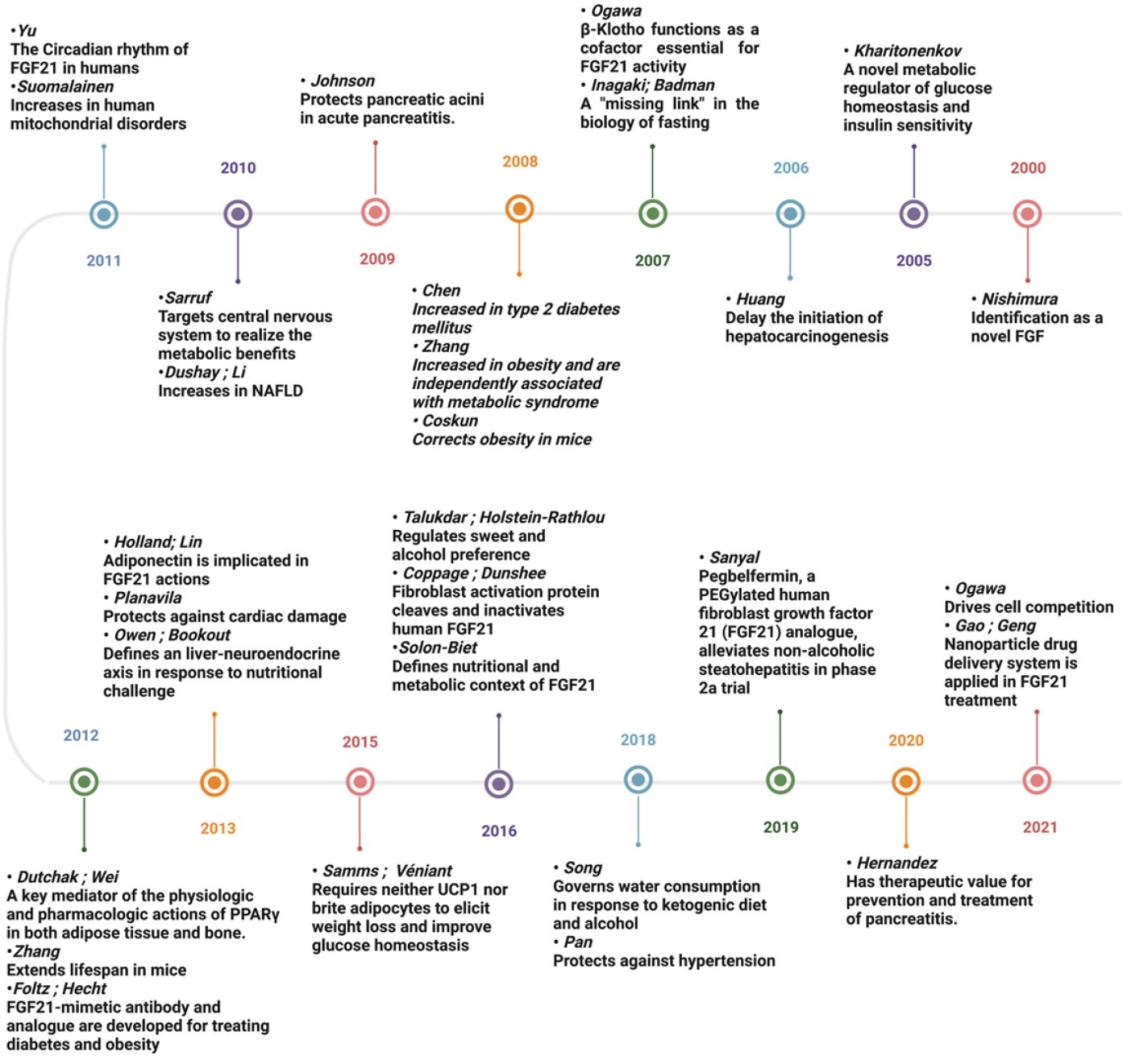
**Timeline figure showing the milestones in the history and development of FGF21.** Since 2000, FGF21 has attracted great interest due to its pleiotropic effects in response to diverse physiological and pathological stress. With the knowledge of FGF21 expanding exponentially, our understanding of its biology is constantly undergoing modification, and the therapeutic value of FGF21 is being hotly discussed.

The mammalian fibroblast growth factor (FGF) superfamily consists of 23 members, which are grouped into 7 subfamilies based on their sequence homology and function. FGF19 (FGF15 for mice) is the only endocrine-acting subfamily that can secrete into the circulation and function as hormones 1. FGF21 is a member of FGF19 family identified in 2000 by Nishimura, Nakatake 2. Over the last two decades, FGF21 has attracted great interest due to its pleiotropic metabolic effects in response to diverse physiological and pathological stress (**Figure 1**). With the expansion of knowledge of FGF21, our understanding of its biology is constantly undergoing modification, especially for the liver-secreted FGF21, which is considered the main source of circulating FGF21 and has been implicated in various diseases (**Table 3**). As a dominant metabolic organ, the liver can sense the stress from both external and internal environments, and accordingly activate its intra- and extra-organ metabolic activities 3. The latter is realized partly via a group of proteins named hepatokines which are exclusively or predominantly secreted from the liver 4. In this regard, there is a growing body of research that points to the role of FGF21, as a newly defined member of hepatokines, in liver-participated inter-organ communications and extra-hepatic diseases. Here, we will discuss current studies and gaps in aberrant expressions and the underlying mechanisms of hepatic FGF21 observed in inter-organ crosstalk and cancer progressions. And in light of the findings summarized in this review, we proposed that FGF21 is a sensitive hormone regulated for adaptive stress response, a potent modulator acting as an endocrine hepatokine in inter-organ crosstalk, and also a promising biomarker with pleiotropic activities in cancer progressions.

## FGF21 is a sensitive hormone regulated for adaptive stress response

Mechanically, the expression and secretion of hepatic FGF21 are coordinately regulated by a set of proteins which are partly listed in **Table 1**. Generally, FGF21 is mainly under the control of peroxisome proliferator activated receptor α (PPARα) as an adaptive response to stress 5. The trafficking and secretion of FGF21 are regulated by the Yip1 domain family member 6 gene, which sorts and packages FGF21 into COPII vesicles for secretion with the guidance of SEC23A 6. As a stress-induced factor, the expression of FGF21 is closely connected with a series of biological events, such as circadian rhythm, diet intervention, exercise, and cold exposure.

### Circadian rhythm

**Figure 2:**
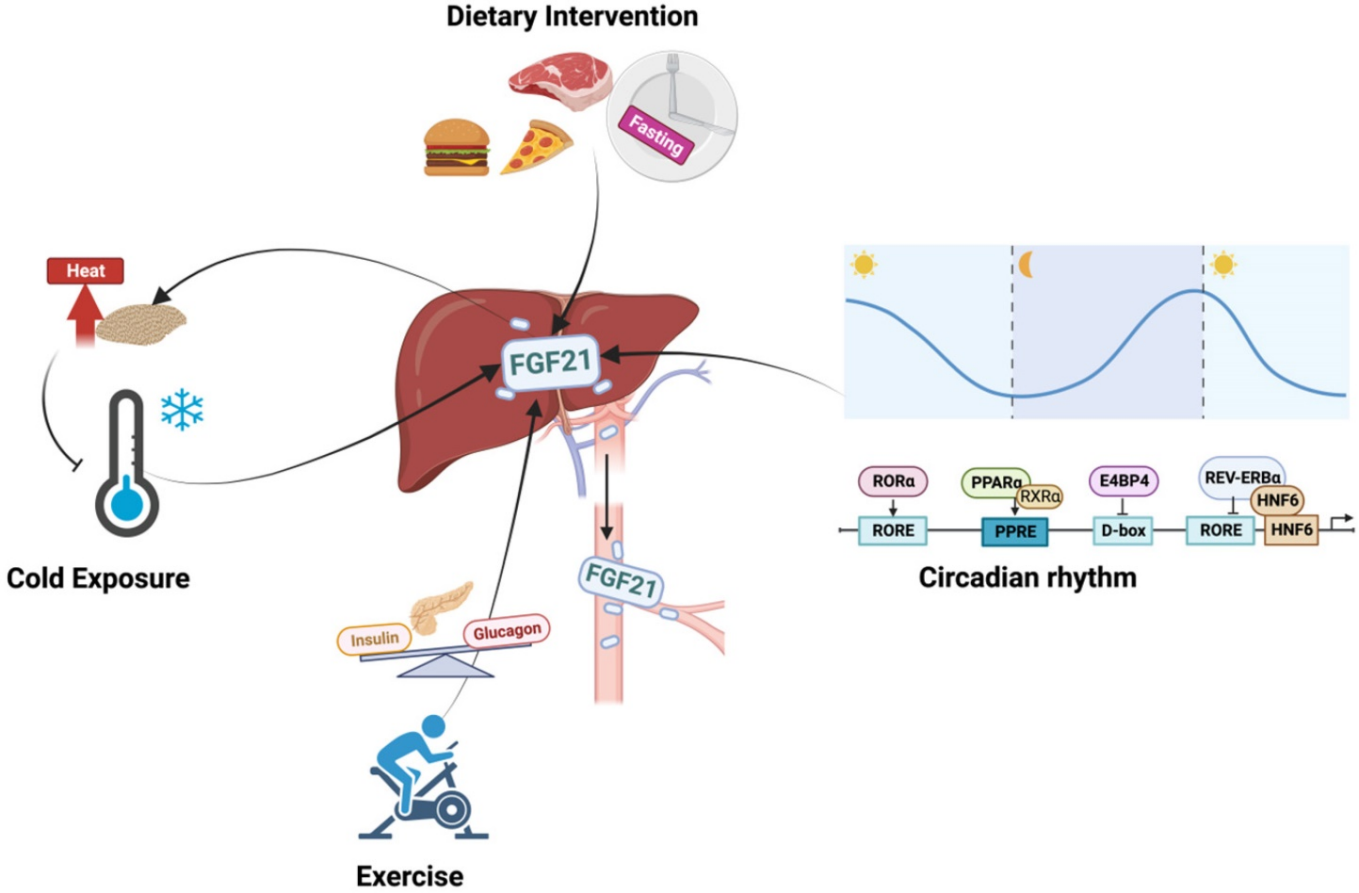
** The regulation of hepatic FGF21.** The expression of FGF21 is tightly connected with circadian rhythm, diet intervention, exercise, and cold exposure. The circadian rhythm of FGF21 is controlled by several clock proteins. PPARα and RORα positively activate the expression of FGF21 while REV-ERBα and E4BP4 repress its transcription; Exercise stimulates FGF21 expression via the increased plasma glucagon to insulin ratio, and this process positively correlated with the elevated lipolysis and decreased glucose levels; Diet regimen has magnificent impacts on FGF21 levels, both nutrient proportions and food timing are implicated in the complex regulation system of FGF21; Increased FGF21 is also observed under cold exposure which may be cooperated with the thermogenic response of BAT to maintain body temperature.

For both humans and mice, serum FGF21 shows circadian rhythm during fasting with the nocturnal rise and diurnal fall, but this pattern is largely diminished in obese individuals and under standardized meals or cold exposure 7-9. As a circadian output gene, the FGF21 promoter contains circadian-responsive elements, and the circadian oscillation of FGF21 is therefore controlled directly by several clock proteins (**Figure 2**) 10-12. Particularly, PPARα is the major activator of FGF21 via binding to PPRE sites during fasting 13, and insulin-mediated upregulation of E4BP4 suppressed FGF21 transcription via D-box under feeding 10. In addition, while RORα activated the FGF21 promoter via an upstream RORE site, REV-ERBα negatively regulated FGF21 expression with the cooperation of HNF6 at RORE site 11, 12. Interestingly, the circadian rhythm of FGF21 seems to be influenced by gender and fed state. Early studies reported that whether under fed or fasting states, both male and female subjects displayed circadian changes 7, 8. But Foo, Aronis 14 later found that the day-night variation patterns of FGF21 only showed in females with standardized meals, but not in fasting state or in male subjects, which were positively correlated with circulating patterns of free fatty acid (FFA) 9, 14. The discrepancy may be secondary to the different study designs, the race of the subjects, and the assays used. However, it indicated that sexual dimorphism exists in the circadian rhythm of FGF21.

### Exercise

For both rodents and humans, exercise resulted in a significant increase in serum FGF21 levels, which were in line with the elevated lipolysis and decreased glucose levels 15, 16. Mechanistically, the upregulated glucagon to insulin ratio during exercise contributed to the increased circulating levels of FGF21, and the concomitant blood FFA elevations may act synergistically on hepatic FGF21 secretion 15. On the other hand, studies found that, under certain conditions, exercise could stimulate FGF21 production in the muscle, which was also associated with the increased circulating FGF21 17. However, divarication was observed in both human and animal-based studies 16, 18. These paradoxical findings suggest that both muscle and liver respond to the exercise-induced elevation of circulating FGF21, but the dominance may change under different conditions. It is noted that the response of FGF21 to exercise is transient, and the sampling time is important. This may explain the lack of difference between the before and 48h after exercise 19. Moreover, different exercise modes and sex also contributed to different outcomes. Studies found that long-term endurance exercise increased FGF21 while neither shortened time of endurance exercise nor resistance exercise changed serum FGF21 levels in men subjects 20, 21. For women, long-term endurance training reduced baseline FGF21 levels, while resistance training significantly increased FGF21 levels 22, 23. Even though these confusing scenarios do lend support to the idea that exercise alters circulating levels of FGF21, further study is warranted to delineate the mechanism explaining the complex patterns of FGF21 facing exercise, and both the study subject and training plan need to be carefully designed to gain more compelling evidence.

### Dietary intervention

The sensitivity and tendency of FGF21 to dietary intervention are different between mice and humans. Both hepatic and circulating levels of FGF21 are robustly induced by fasting, high-fat diet, low-carbohydrate, and high protein ketogenic diets in mice 5, 24. However, humans showed a more complicated response to diet intervention. Circulating FGF21 levels are not altered in humans by either shorter-term fasting or ketogenic diets, but increased by long-term fasting (7 days) or with a low-protein/high-carbohydrate diet 7, 25-27. Besides, human FGF21 was sharply increased after 3-day overfeeding with high-fat snacks but almost returned to baseline on day 28 28. These findings indicated the complex intrinsic variation in FGF21 metabolism, which is regulated by both fasting and feeding signals. Moreover, a sex-dependent difference was also implicated where female mice were less sensitive than males to metabolic improvement observed following the high-carbohydrate diet corresponding with the less significant increase in circulating FGF21 level, but this difference was largely reduced after ovariectomy 27. In view of the sexual dimorphism observed above, we can speculate that the metabolic benefits of FGF21 for diet intervention among females are regulated by ovarian steroid hormones (or the intact female reproductive system), though further study is required to reveal the underlying mechanisms.

### Cold

While increased FGF21 levels, decreased half-life, and retained diurnal rhythm during cold exposure have been consistently observed in mice 29, changes in humans are still under debate. Paul Lee *at el*. 30 and Hanssen *at el*. 31 found that mild cold exposure augmented overall FGF21 levels, which were positively associated with improved body core temperature. However, some studies observed decreased plasma FGF21 levels in either short-term mild cold exposure or overweight subjects 32, 33. It should be noted that the diurnal rhythm of plasma FGF21 was weakened but still retained under mild cold exposure 34.

On the other hand, FGF21 expanded overall thermogenic capacity by stimulating fat browning activity 30. Therefore, brown adipose tissue (BAT) was also implicated in the regulation of FGF21 under thermogenic activation 29, 35. Norepinephrine-stimulated increase of FGF21 outputted from interscapular BAT caused marked arteriovenous differences in FGF21 levels under cold exposure 29. However, counterevidence has also been reported 35, 36. In FGF21 liver-specific-KO mice, both acute cold exposure and norepinephrine-induced increases in circulating FGF21 levels were completely lost, accompanied by the markedly attenuated BAT sympathetic nerve activity and impaired BAT glucose metabolism 37, 38. These findings support a more plausible hypothesis that cold exposure evokes the simultaneous elevation of FGF21 in the liver and BAT, wherein the BAT-divided FGF21 shows autocrine and paracrine effects on activating the thermogenic response of BAT, while hepatic FGF21 modulates the circulating FGF21 levels and signals to the central nervous system (CNS) to enhance this adaption function further.

The regulation of FGF21 is complex and still not being fully understood. In this regard, establishing strict eligibility criteria for participant selection, measuring FGF21 levels at multiple time points, and setting paired samples are indispensable for studies to decipher the precise response of FGF21 under certain conditions.

## FGF21 is a potential modulator in liver-participated inter-organ crosstalk

**Figure 3:**
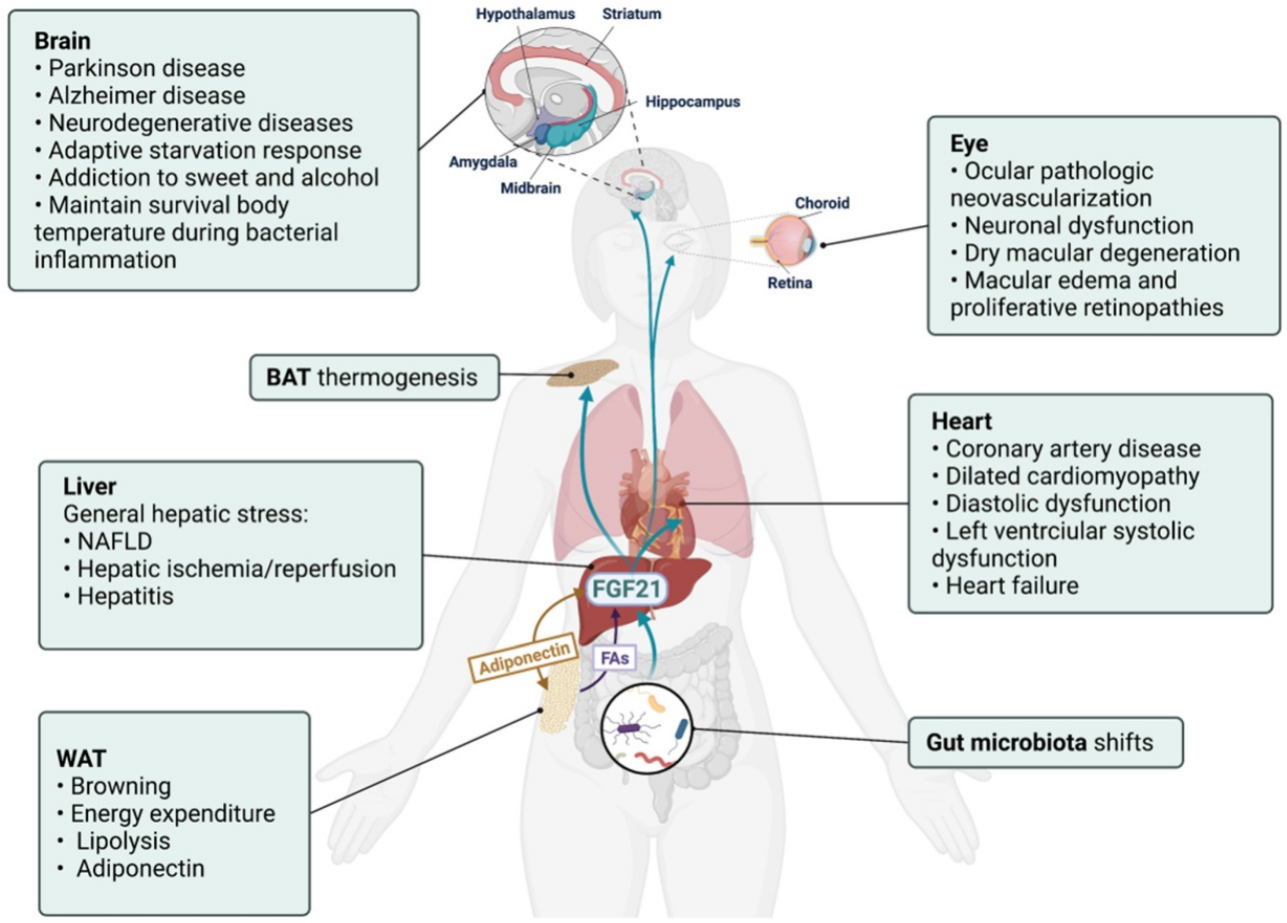
** Hepatic FGF21 is a modulator in liver-participated inter-organ crosstalk.** The aberrant expressions of FGF21 have been associated with brain, eye, and heart diseases and implicated in various liver-participated inter-organ crosstalk. As a modulator, hepatic FGF21 has displayed an important role in mediating brain-regulated adaptive response and adipose tissue-performed energy metabolism.

FGF21-FGF receptors (FGFR) axis could activate a multitude of signaling cascades, including the RAS-MAPK pathway, the JAK-STAT pathway, the PI3K-AKT pathway, the PLCγ pathway, and JNK pathways 39. As a pleiotropic metabolic regulator, the activities of FGF21 are complex and vary in different physiological or pathophysiological context 40, and whether the liver is the direct target of FGF21 remains debatable. Hepatocytes express β-Klotho (KLB) but not FGFR1. Therefore,* in vivo* administration of FGF21 stimulated the response of white adipose tissue (WAT) and breast tissue but not the liver 41. However, FGF21 enhanced hepatic fatty acid oxidation and tricarboxylic acid cycle flux 42, implicating that FGF21 exerted these hepatic actions via an indirect mechanism that may involve other secretory factors. On the other hand, as a secretory hepatokine, hepatic FGF21 is supposed to integrate liver metabolic status with systemic needs and may contribute to inter-organ communications. The aberrant expression levels of circulating FGF21 in different diseases (**Table 3**) show its pleiotropic effects. Up to now, the role of FGF21 has been unearthed in several organs/tissues, and the existence of FGF21-based inter-organ crosstalk was implied (**Figure 3**).

### Nervous system

Though undetectable in the nervous system, FGF21 could cross the blood-brain barrier and act in the CNS 43. Moreover, FGF receptors FGFR1c, 2, and 3c are broadly expressed in the nervous system, and co-receptor KLB is selectively expressed in the suprachiasmatic nucleus 44. Hepatic FGF21 regulated glucocorticoid production via the CNS 45. Also, FGF21 has defined an important liver-neuroendocrine axis to modulate the adaptive starvation response, including the regulation of circadian behavior, body weight, and insulin levels via suppressing the output of the suprachiasmatic nucleus 44. Similarly, the caloric restriction increased hepatic expression and systematic concentration of FGF21, and FGF21 treatment showed neuroprotection effects via activating FGFR1 pathways in primary glial cells 46. An intriguing study about gut microbiota transplantation found that the increased expressions of hepatic/systemic FGF21 and hippocampal KLB expression were positively connected with improved neurogenesis in recipients, shedding light into the link between a FGF21-based microbial and the neuron system 47.

There has been a focus on neuroprotection activities of FGF21 in brain disorders in recent years. Decreased serum FGF21 levels were detected in patients with Parkinson's disease and Alzheimer's disease 48, 49. And FGF21 treatment improved related neural pathologies and degeneration by rescuing the astrocyte-neuron lactate shuttle system 50-52. In this regard, the FGF21-expressing lentiviral vector and FGF21 analogue LY2405319 have been proposed as promising therapy for Alzheimer's disease 53, 54. However, whether these neuroprotective functions are related to the hepatic secretory FGF21 has not yet been reported. Instead, the hippocampus and cortex could secret FGF21 under neuronal mitochondrial dysfunction 55, and an increased pancreas source of FGF21 was also reported to improve CNS damage 56. Therefore, the aberrant FGF21 levels detected in neuronal diseases (especially in conditions associated with the neuron mitochondrial and endoplasmic reticulum stress) in both brain and blood should be identified with caution, and the liver-CNS axis may help further explore the neuroprotection activities of FGF21.

### Gut microbiome

Gut microbiota (GM) represents a mysterious group of microorganisms living in the digestive tract, and host-GM crosstalk has been discussed in various human diseases 57. The FGF19-bile acid-GM axis has been established and implicated in the GM-organs crosstalk, especially with the liver 58. Hepatic FGF21 expression is influenced by GM and involved in the microbiota-gut-brain axis 47. Moreover, decreased serum FGF21 levels were detected in *Clostridioides difficile* infection patients receiving fecal microbiota transplantation therapy 59. Mice supplied with *B. adolescentis*, the dominant species in the human intestine, showed reduced hepatic FGF21 levels, which were believed as an improvement of the non-alcoholic fatty liver disease (NAFLD)-related FGF21 resistance state 60. More compelling, it was found that the increased hepatic FGF21 expression of protein restriction relied on the activity of GM, and dietary fiber supplementation influenced hepatic FGF21 stress response via shifting GM composition 61. However, the significance of GM-induced hepatic FGF21 response to bodily functions, and whether the GM-liver communication is bidirectional requires further exploration.

### Adipose tissue

While the liver is the major contributor to circulating levels of FGF21, WAT is the target contributing greatly to the metabolic effects of FGF21. The expression of KLB in conjunction with FGFR1c in adipose tissue confers FGF21 activities there 62, 63. The FGF21 expression level in human adipose tissue is negligible 25, which indicates that liver-secreted FGF21 is requisite for realizing the metabolic benefits of FGF21 found in adipose tissues. Along this line, studies have revealed the important role of FGF21 in liver-adipose tissue crosstalk. The elevated liver production of FGF21 in hepatic Cpt1a knockout mice contributed to the increased adipose browning and energy expenditure 64. A recent study found that methionine adenosyltransferase *Matla* knockdown prevented and reversed obesity, insulin resistance, and hepatosteatosis via stimulating the expression and secretion of hepatic FGF21, which subsequently activated the liver-adipose tissue axis resulting in increased BAT thermogenesis, WAT lipolysis, and secretion of adiponectin 65.

The interactions between FFA and FGF21 create the loop between WAT and the liver. The FFA level in the serum of healthy individuals showed a similar oscillatory pattern with FGF21 under both fasting and feeding conditions, though the pick time was preceded. On the one hand, hepatic p38 stimulated WAT lipolysis and FFA release via upregulating FGF21 secretion from the liver to WAT 66. On the other hand, a low dosage of FFA treatment stimulated FGF21 secretion in HepG2 cells 8. And growth hormone-stimulated release of FFA from WAT resulted in the CREBH/PPARα-mediated over-expression of hepatic FGF21, which in turn suppressed WAT lipolysis 67, 68. Notably, a study found that only unsaturated FFA could induce hepatic FGF21 expression 8. Considering the FFA composition is different in different sites of WAT 69, it is worth uncovering whether their effects on hepatic FGF21 are also site specific.

Adiponectin is the most abundant adipokine secreted by adipocytes 70. Intriguingly, recent studies have started to delineate the positive interactions between FGF21 and adiponectin. A study found that hepatic FGF21 induced the biosynthesis and secretion of adiponectin in adipocytes, which in turn conferred FGF21 systematic and hepatic functions through positive feedback 71. And WAT adiponectin was reduced in FGF21-KO mice but was completely restored after FGF21 replenishment 72. Furthermore, both acute and chronic administration of FGF21 increased circulating adiponectin levels, and adiponectin deficiency diminished the metabolic effects of FGF21 on glucose tolerance and insulin resistance 71. Magnificently, the study found that JNK deficiency-induced adipocyte FGF21 upregulation triggered a feed-forward loop in the liver-WAT axis, which promoted hepatocyte FGF21 expression and secretion via regulating adipocyte adiponectin expression and secretion 73. These findings represent an unfolding story about the interactions between FGF21 and adipokine.

### Heart

The evaluated levels of FGF21 were predictive of poor outcomes in cardiac diseases 74, 75 (**Table 3**). Interestingly, one recent study detected that the cardiac FGF21 gene expression levels in heart failure patients were as low as healthy subjects, but the protein levels of FGF21 in both the serum and cardiac section were aberrantly increased and accompanied by elevated cardiac FGFR3 expression, which indicated the existence of a FGF21-dependent cardiohepatic signaling circuit 74. Consistently, hepatic FGF21 maintained chronotropy in mice during bacterial inflammation 76. Mice cardiomyocytes express FGFRs and KLB, and the activated PI3K-Akt and ERK signaling pathways partly contributed to the cardiac benefits of FGF21 77, 78. However, the mechanism of FGF21 cardioprotective actions is still under debate, and the existence of the FGF21-based liver-brain axis remains to be proved. For example, cardiomyocytes were not only a target but also a source of FGF21 78. KLB expression was diseased in cardiac dysfunction, and *in vivo* FGF21 treatment failed to activate ERK signaling in the heart 76. These findings indicated that both systemic and cardiac FGF21 had cardioprotective activities via direct, indirect, or both pathways.

### Retina /Choroid

Retinal and choroidal ocular pathologic neovascularization is aggravated in FGF21 knockout mice and suppressed via the FGF21 analog-activated adiponectin pathway 79. FGF21 depletion also resulted in the increased occurrence of dry macular degeneration-like pathological changes, while exogenous FGF21 administration reversed this phenomenon 80. A later study found that FGF21 prevented VEGF-induced retinal vascular leakage in mice and strengthened human primary retinal microvascular endothelial cells barrier function 81. However, it is still unclear whether circulating or local FGF21 contributed to these protective effects. Considering the extremely low/absent expression of FGF21 detected in the visual system 82, it is plausible to postulate that the protective effect of FGF21 on the eye is mainly realized by the circulating FGF21, though further studies are necessary.

The data discussed above indicate that the aberrant expressions of FGF21 observed in circulation and organs in different contexts are likely to be an ex officio action of the liver to realize the pleiotropic effects of FGF21, especially regarding the adaptive starvation response in the CNS system, nutrition metabolism in GM, and energy homeostasis in adipose tissue. A crosstalk network is therefore delineated between the liver and other organs/tissues, which is no doubt striking and should be an area of fruitful future study.

## FGF21 is a promising biomarker with pleiotropic activities in cancer progressions

**Figure 4:**
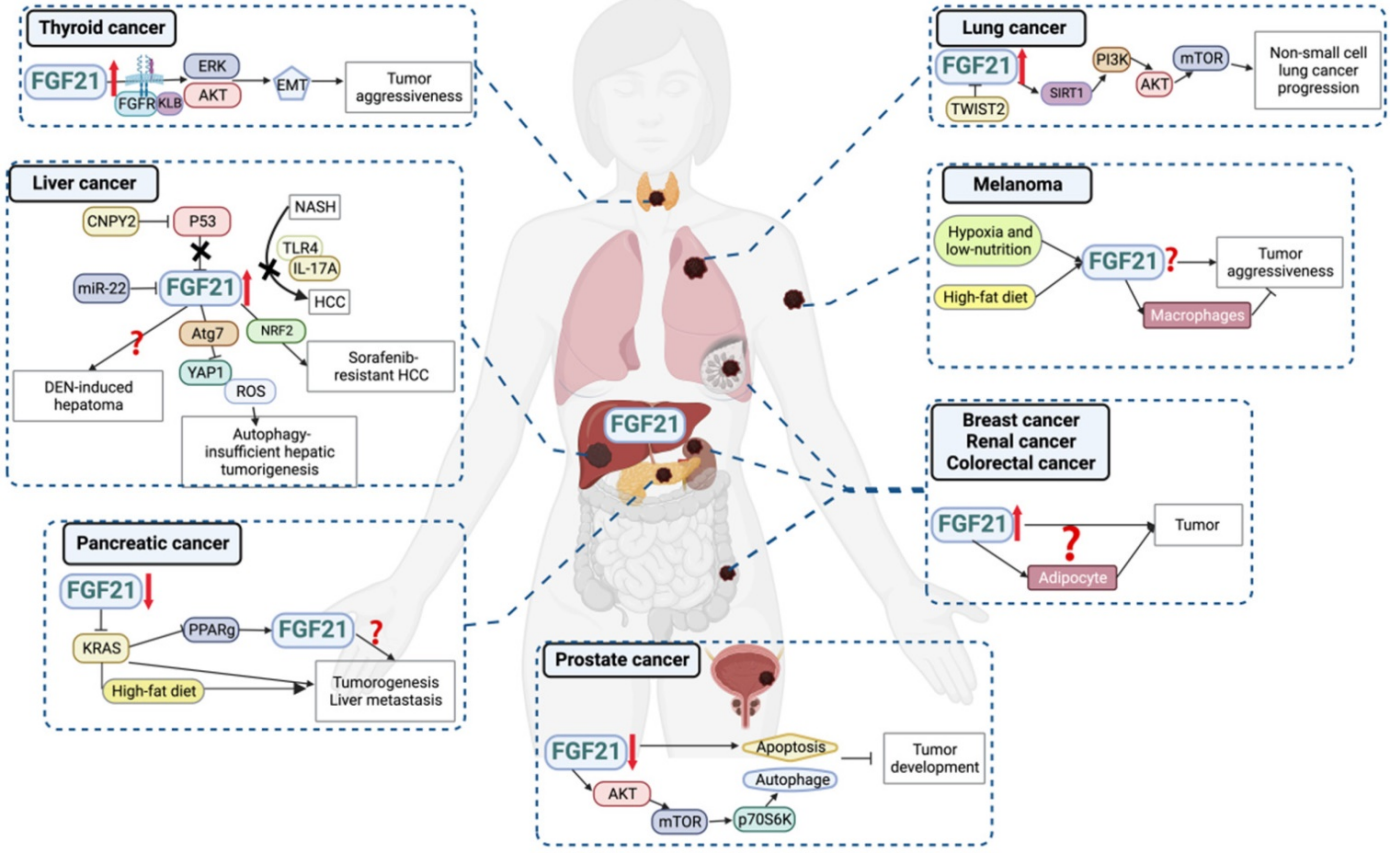
** Hepatic FGF21 is a promising biomarker for cancers.** Aberrant expression of FGF21 has been correlated with cancer development. Accumulating data indicated the predictive and diagnostic values of FGF21 as a promising biomarker for human cancers, for some of which its underlying mechanisms have been discussed.

As a stress-responsive hepatokine, the implications of FGF21 in cancers have been focused on in recent years. Overall, overexpression of FGF21 in both circulating and tissue levels has been found in different types and clinical stages of cancers (**Figure 4, Table 2**), suggesting the biomarker value of FGF21 in cancer diagnosis and treatment. Theoretically, the well-studied findings of the oncogenic FGF-FGFR axis attest to this hypothesis. FGF21-related FGF receptors FGFR1c, 2c, 3c, and 4 are widely expressed in tumor cells and aberrantly activated in cancers 83. The deregulation of FGF expression drives oncogenic FGFR signaling and activates downstream signaling through MARK-ERK, PI3K-AKT, and JAK-STAT pathways that regulate tumor cell proliferation, differentiation, and survival 84. Another plausible reason for FGF21 participating in cancers is its high sensitivity to macronutrients. Imbalanced nutrition intake is not only a cause of carcinogenesis but also occurs during cancer progression. In this setting, recruiting FGF21 from either adjacent or distant organs will benefit tumor tissues against adverse environments. This may partially explain the elevated circulating and peritumoral FGF21 levels observed in cancer patients 85, 86. However, in a low-protein ketogenic diet, FGF21 knockout mice showed a comparable decrease in tumor growth to that of the wild type 87. Therefore, although the aberrant expression of FGF21 was a sensitive response to diet intervention, whether it benefits anti-cancer activities in low protein diets and ketogenic diets or plays a part in countertrending obese diets requires further study 88.

### Liver cancer

The close relationship between the liver and FGF21 aroused researchers' attention to the role of FGF21 in liver cancers. Accumulated data gave us a relatively deeper understanding of the FGF21-liver cancer axis compared to other cancers. Though FGFR4 is the major FGFR present in mature hepatocytes, elevated expression of FGFR1 has been observed in hepatocellular carcinoma (HCC) and contributed to tumor development 89. This aberrant expression appears to be more favorable for FGF21's binding. Consistently, both liver and serum levels of FGF21 were upregulated in patients with hepatitis, cirrhosis, and liver tumors, and associated with a significantly decreased survival rate in patients with inferior hepatocellular carcinoma 90. For the carcinogen-induced HCC animal model, increased FGF21 levels were observed in the early and middle stages of tumorigenesis, and also in normal hepatocytes adjacent to the tumor foci, while sharply diminished once cells progressed to malignancy. This change indicated that FGF21 is an independent indicator of genetic hepatocarcinogenesis, but its expression is not a direct genetic marker of hepatoma cells per se 91. Instead, a study showed that canopy homolog 2 promoted liver oncogenesis via destabilizing p53 and activating hepatic FGF21 expression 92. Consistently, tumor suppressor *miR-22* reduced hepatic FGFR1 by direct targeting and inhibited FGF21 expression by reducing its synthesis process 93. Moreover, FGF21 showed a positive feedback loop with NRF2 accounting for sorafenib's resistance. Higher FGF21 expression was correlated with more resistance to sorafenib treatment in HCC patients, and intra-tumoral FGF21 knockdown effectively inhibited tumor growth in sorafenib-resistant HCC animal models 94. These findings indicated that the pathological upregulation of FGF21 promoted tumor development.

On the other hand, some studies have discussed the tumor suppressor effects of FGF21 on HCC under both physiological and pharmacological levels. FGF21 knockout mice were more sensitive to a high fat, high sucrose diet-induced HCC 95, which was later proven through FGF21's negative feedback on hepatocyte-TLR4-IL-17A signaling 96. And FGF21 knockout accelerated autophagy gene *Atg7* deletion induced hepatic tumor process, implicating the suppressive effect of FGF21 on autophagy-insufficient hepatoma 97. Besides, the overexpression of FGF21 delayed the tumor incidence in the diethylnitrosamine-induced liver cancer mice model, but contrarily accelerated the progression of adenoma to HCC, resulting in similar HCC outcomes between overexpression and wild-type groups 98. Also, the distinct effects of long-term pharmacological dosage of FGF21 on HCC were observed between rats and mice 99, indicating that the pharmacological effects of FGF21 on hepatoma may be highly susceptible to species and treatment regimens.

### Thyroid cancer

Serum FGF21 levels were significantly higher in the papillary thyroid carcinoma patients than in controls and were positively associated with pathological stage, recurrence, and mortality. *In vitro* FGF21 treatment promoted tumor cell migration and invasion by upregulating FGFR-EMT signaling. It is worth noting that FGF21 expression was detected in neither normal thyroid tissues nor tumor tissues, which indicated that FGF21 might induce thyroid tumor progression in an endocrine way 85.

### Prostate cancer

FGF21 expression was decreased in clinic prostate cancer tissues, and overexpression of FGF21 inhibited prostate cancer cell viability 100. But whether the decreased FGF21 levels in tumor tissues were due to the decreased circulating FGF21 needs to be confirmed. Besides, using high glucose conditions to strengthen cell viability seems uncalled for considering cancer cells' commonality of uncontrolled proliferation and anti-apoptosis abilities. Furthermore, it may lead to the effects of FGF21 on cancer cells easily overshadowed by its outstanding regulation ability of glucose metabolism, which the author also mentioned.

### Non-small cell lung cancer

FGF21 was upregulated in non-small cell lung cancer tissues, and the higher expression level was associated with shorter overall survival time and advanced histological type. *In vitro* study showed that FGF21 was elevated in tumor cells compared with normal lung cell lines, which facilitated tumor progression by promoting cell growth, migration, and defending oxidative stress via Sirtuin 1/PI3K/AKT signaling 86. Moreover, FGF21 expression was suppressed in TWIST2-overexpressed lung cancer cells, resulting in decreased cell viability, increased oxidative stress, and cell apoptosis 101. However, current research only focused on the autocrine function of FGF21 in lung cancers. Thus future studies concentrating on the interplay between the endocrine form of FGF21 and lung tumor tissues are encouraged.

### Melanoma

Overexpression of FGF21 in mouse melanoma cell line enhanced cell aggressiveness and tumorigenicity under hypoxia and low-nutrition double deprivation stress 102. However, FGF21 serum levels were negatively connected with the metastatic ability of melanoma cells in animals, and exogenous FGF21 treated macrophage inhibited melanoma cell viability via intercellular crosstalk in a dose-dependent manner 103. This controversy reflected the pleiotropic actions of FGF21 in melanoma and the tumor microenvironment.

### Pancreatic cancer

The circulating levels of FGF21 in pancreatic cancer patients are still unclear, but both pancreatic cancer specimens and cell lines had lower levels of FGF21 than normal human pancreatic tissues and cells, respectively. The pharmacological dosage of FGF21 supplementation effectively inhibited pancreatic intraepithelial neoplasia lesions, inhibited liver metastasis, and prolonged the overall survival of KRAS-mediated pancreatic cancer under the high-fat-diet challenge. Though serum FGF21 level was not influenced by pancreatic KRAS mutation, the effect of pathological levels of circulating FGF21 under a high-fat diet, in turn, on pancreatic tumor development was not addressed 104. Moreover, KRAS, the most frequently mutated RAS isoform, happens not only in pancreatic cancer but also in lung cancer, multiple myeloma, and colorectal cancer 105. Whether this KRAS-FGF21 axis exists in other types of cancer and the distinctions between different mutant hotspots deserve further study.

### Breast cancer, Renal cancer and Colorectal cancer

As mentioned, adipose tissue is not only a source of autocrine FGF21 but also a direct and predominant target of hepatic FGF21, which may result in a substantial enrichment of FGF21 in the microenvironment of the tumors growing in the anatomical vicinity of adipose tissue, such as breast, renal and colon cancers. Even though the activities of FGF21 in these types of cancers have not been explored yet, elevated serum levels of FGF21 were reported in the early stages of breast cancer 106 but significantly reduced following 12 months of hormonal therapy 107. Patients with both clear renal cell carcinoma and chromophobe renal cancer had higher levels of serum FGF21 compared with healthy control 108. Also, FGF21 has shown positive associations with colon site cancer, stage I-II colorectal cancer, advanced colorectal neoplasia, and even in cases collected over 5 years before diagnosis, which indicated that FGF21 is potentially predictive and possibly existing in colorectal cancer etiology 109-111. Compatible with these findings, higher circulating levels of FGF21 increased the risk of metachronous colorectal adenomas, especially in the older population 112. These limited data suggest the great suitability of serum FGF21 as a diagnostic biomarker of cancers, and also open the door for the speculation that an FGF21-driven liver-adipose tissue-tumor tissue axis is implicated in cancers.

### Other cancers

Increased FGF21 levels were observed in patients with different stages of gastric cancer, suggesting it is a suitable biomarker for early-stage gastric cancer 113. Also, serum FGF21 level was higher in patients with endometrioid carcinoma, sufficing it to diagnose the cancer stage and grade 114. Similarly, in urothelial carcinoma, serum FGF21 level was positively associated with the tumor stage, cardiovascular disease, and history of recurrence 115.

So far, there is limited information available to explain the role of FGF21 in tumor initiation and progression. Though the results listed above are promising, they need more detailed and in-depth investigations as it is hard to discern whether the increased level of FGF21 contributes to cancer development or is just a result of carcinogenesis or a stress response for maintaining body system homeostasis. As a potential biomarker, the aberrant expression of FGF21 has been generally observed in different types and stages of cancers, which indicates its low specificity. Instead, FGF21 may be an ideal non-invasive biomarker applied in regular health tests for discovering and preventing cancers at an early stage, though the sensitivity and cancer-specificity for clinical use need to be evaluated critically.

## Discussion

Since 2000, the scientific appreciation of FGF21 in various human diseases has been rapidly expanding. As an unfolded area, the increasing roles of FGF21 generate a series of inspiring ideas, such as whether exclusive FGF21 secretion ability confers liver extra-hepatic functions, whether endocrine FGF21 is the holistic hub point for interorgan crosstalk, whether there are concomitant modulators that collaborate with FGF21 or whether FGF21 is an actionable biomarker with therapeutic values for cancers. However, studies in this area are still in their infancy. To boost the research process of FGF21, several factors need to be considered.

The first factor is about distinguishing between pathological and pharmacological doses of FGF21. The physiological actions of FGF21 occur at much lower concentrations and in more restricted organ systems and tissues than its pharmacological actions 40. Both long-term administrations of pharmacological dosage of FGF21 and transgenic mice over-expressing FGF21 did not develop liver tumor or show evidence of any other tissue hyperplasia, which potently denied the carcinogenic risk of FGF21 therapy 116, 117. Future studies regarding the role of aberrant pathological levels of FGF21 in cancer progression are therefore recommended. The distinction between pathological and pharmacological levels of FGF21 did not receive adequate attention in previous studies. The highly inconsistent dosage selection was observed in studies that were all focused on the pharmacological actions of FGF21 99, 104. Furthermore, converting *in vitro* concentrations to *in vivo* doses (and vice versa) is even thornier since it is hard to set a clear line between pathological and pharmacological dosages for *in vitro* study. In this case, *in vivo* study is more reliable for observing the biological effects of FGF21 under different conditions.

Secondly, the elimination half-life of FGF21 is only 0.5~2 hours 116, 118. Thus selecting the appropriate administration dosage and mode is important and highly dependent on the objective of your research. Even though a study indicated that FGF21 action did not require the prolonged presence of the actual protein in circulation, approximately a 10-fold greater dose of FGF21 was needed for a once-daily subcutaneous injection to achieve an equivalent body weight reduction compared with continuous FGF21 administration via osmotic pump 116. Since continuous infusion allows the stable presence of circulating FGF21 levels throughout the course of the study, it is particularly suitable for studying the long-term effects of FGF21 (such as mimicking the overall increased FGF21 levels in metabolic diseases or testing the chronic therapeutic efficacy of FGF21). Furthermore, the bioactivity of recombinant FGF21 protein should also be evaluated with caution. It should be realized that different purification and activity, combined with the non-standardized selection of dosage and administration mode, will leave non-negligible impacts on the results and further contribute to the inconsistencies between studies.

Besides, the similarities and differences between mice and humans need to be considered. While it may sound hypercritical, the aforementioned distribution and activity difference between human and mouse FGF21 warn us of the existence of species specificity in various contexts. Therefore, the clinical translation of FGF21 activities demonstrated in mice to humans needs to be further investigated. For example, mouse adipose tissues express detectable levels of FGF21, which has been implicated in the magnificent effects of FGF21 on energy metabolism. However, human adipose tissues do not express FGF21, which questions the clinical value of autocrine activities of adipose FGF21 observed in rodent-based studies 119. Also, studies found that a significant percentage of circulating human FGF21 is not active due to fibroblast activation protein-mediated post-translation and inactivation. In contrast, mouse FGF21 is resistant to cleavage because of the different C-terminus 118, 120. The conjunction of these overlooked differences may finally contribute to the lower-than-expected translation efficacy of FGF21-based therapeutic benefits from bench to bedside 121.

Last but not least, it is plausible that the physiology and function of FGF21 may be sexual dimorphism considering the substantial difference between genders in sex hormone levels and macronutrient metabolism patterns. Besides the different sensitivity to circadian rhythms 9, 14, exercise 20, 21, and diet 27, pharmacological levels of FGF21 showed stronger metabolic effects in males and were antagonized by estradiol replenishment in ovariectomized females 122, 123. However, endogenous hepatic FGF21 transcription is positively regulated by the estradiol-Wnt pathway in a PPARα-independent way, which resulted in reduced hepatic FGF21 production in female mice following ovariectomy 124. These findings suggest a notable role of estrogen in FGF21 activities. Sexual dimorphism is a new frontier in FGF21 research, so future studies concentrating on this difference, especially unearthing the orchestrated interactions between FGF21 and estrogen, will expand our understanding of FGF21 biology.

## Conclusion

Focusing on FGF21, the findings discussed in this review indicated its high sensitivity to adaptive stress response, its important role in modulating liver-participated inter-organ crosstalk, and its potential value in cancer diagnosis and treatment. In-depth studies to establish a comprehensive framework of the biology and activities of endocrine FGF21 are called upon.

## Supplementary Material

Supplementary information.

## Figures and Tables

**Table 1:** The direct regulators of hepatic FGF21

Regulator	Condition	Correlation with FGF21 production	Reference
Peroxisome proliferator activated receptor	Gluconeogenesis; Ketogenesis; Adaptive starvation response	+	5
Farnesoid X receptor	Ketogenic diet	+	125
cAMP-responsive element-binding protein H	Fasting; High-fat diet	+	126
Nuclear receptor subfamily 4 group A member 1/Nur77	Fasting	+	127
Activating transcription factor 4	High-fat diet; Carbon monoxide induction; Glucagon plus insulin induction	+	128-130
Carbohydrate responsive-element binding protein	High-fructose diets	+	131
Aryl hydrocarbon receptor	2,3,7,8-tetrachlorodibenzo-p-dioxin-induced hepatotoxicity	+	132
Glucocorticoid receptor	Dexamethasone induction; Glucocorticoids induction	+	45, 133
Liver X receptor	Cholesterol-enriched diet	-	134
Estrogen-related receptor γ	Hepatic CB1 receptor-mediated induction	+	135
E4 binding protein 4	Refeeding	-	136
Yip1 domain family member 6 gene	Trafficking and secretion	-	6

**Table 2:** The correlations and activities of FGF21 in cancers

Cancer	Clinical study		*In vivo* study	*In vitro* study	Reference
	Correlation with circulating FGF21 levels	Correlation with intra-tumoral FGF21 levels			
Liver cancer	+	+	Serum FGF21 levels were different in different stages of tumorigenesis; Intratumoral FGF21 knockdown inhibited sorafenib-resistant HCC; Deficiency of FGF21 promoted obesogenic diet-induced HCC; FGF21 knockout promoted autophagy-deficient hepatoma; Overexpression of FGF21 or exogenous FGF21 treatment delayed carcinogen-induced HCC tumorigenesis; FGF21 treatment promotes carcinogen-induced HCC tumorigenesis in rats.	Overexpression of FGF21 increased sorafenib resistance; FGF21 treatment suppressed carcinogen-induced oxidative stress.	89-91, 94, 95, 97, 99
Thyroid cancer	+	Undetectable	Unknown	FGF21 treatment promoted tumor cell migration and invasion.	85
Prostate cancer	Unknown	-	Overexpression of FGF21 inhibits prostate cancer tumorigenesis.	Overexpression of FGF21 inhibits prostate cancer cell viability.	100
Non-small cell lung cancer	Unknown	+	Unknown	Overexpression of FGF21 promoted tumor cell growth and migration, while down-regulated FGF21 presented opposite effects on lung cancer cells.	86, 101
Melanoma	Unknown	Unknown	Overexpression of FGF21 enhanced tumorigenesis; Serum FGF21 levels were negatively connected with the metastatic ability.	Overexpression of FGF21 enhanced cell aggressiveness and tumorigenicity; Conditioned medium from exogenous FGF21 treated macrophage inhibited melanoma cell viability.	102, 103
Pancreatic cancer	Unknown	-	Pharmacological supplementation of FGF21 delayed cancer development under obesogenic diet challenge.	Decreased FGF21 levels in pancreatic cancer cells.	104
Breast cancer	+	Unknown			106
Renal cancer	+	Unknown			108
Colorectal cancer	+	Unknown			107-109, 112
Gastric cancer	+	Unknown			113
Endometrioid carcinoma	+	Unknown			114
Urothelial carcinoma	+	Unknown			115

**Table 3:** FGF21 is a promising biomarker for risk prediction and diseases progression

Disease	Correlation with circulating FGF21	Reference
NAFLD	+	137, 138
Hepatic ischemia/reperfusion injury in patients with liver transplantation	+	139
Genotype-4 chronic hepatitis C	+	140
Advanced fibrosis/cirrhosis in chronic hepatitis B patients on antiviral treatment.	-	141
Carotid atherosclerotic diseases	+	142
Coronary artery disease	+	75, 143
Heart failure	+	74
Vascular calcification	+	144
Adverse cardiovascular events in patients with type 2 diabetes	+	145
Adverse cardiovascular events in ST-segment elevation myocardial infarction patients	+	146
Adverse clinical events in patients with myocardial infarction who have undergone coronary artery bypass graft	+	147
Diabetes	+	148
Obesity	+	149
Diabetic nephropathy	+	150
Renal disease	+	151
Mitochondrial disease/mitochondrial myopathies	+	152
Pediatric mitochondrial disease	+	153
Primary sarcopenia	+	154
Dravet syndrome	+	155
Pterygium	-	156
Missed abortion	+	157
Mortality of patient with sepsis and acute respiratory distress syndrome.	+	158

## Abbreviations

FGF: fibroblast growth factor
PPARα: peroxisome proliferator activated receptor α
BAT: brown adipose tissue
CNS: central nervous system
WAT: white adipose tissue
GM: gut microbiota
COPII: coat Protein Complex II
RORα: retinoic acid-related orphan receptor alpha
HNF6: hepatocyte nuclear factor 6
FFA: free fatty acid
FGFR: fibroblast growth factor receptor
MAPK: mitogen-activated protein kinase
JAK: Janus kinase
STAT: signal transducer and activator of transcription protein
PI3K: phosphoinositide 3 kinase
AKT: serine/threonine-protein kinase
PLCγ: phospholipase C gamma
JNK: c-Jun N-terminal kinase
KLB: β-klotho
NAFLD: non-alcoholic fatty liver disease
VEGF: vascular endothelial growth factor
ERK: extracellular signal-regulated kinases
HCC: hepatocellular carcinoma
TLR4: toll-like receptor 4
IL-17A: interleukin 17A
Atg7: autophagy related 7
EMT: epithelial-mesenchymal transition
TWIST2: twist family BHLH transcription factor 2
KRAS: kirsten rat sarcoma virus
RAS: rat sarcoma virus
