# Supplementary material for: Hepatic FGF21: Its Emerging Role in Inter-Organ Crosstalk and Cancers
Source: Int J Biol Sci. 2022 Oct 3;18(15):5928–42. doi: 10.7150/ijbs.76924 (PMC9576513; doi:10.7150/ijbs.76924)
Supplement: Supplementary file 1 — Supplementary information. [file ijbsv18p5928s1.pdf]

Supplementary information for Figures:

1. References for Figure 1

Figure 1 Timeline figure showing the milestones in the history and development of FGF21

Since 2000, FGF21 has attracted great interest due to its pleiotropic effects in response to diverse physiological and pathological stress. With the knowledge of FGF21 expanding exponentially, our understanding of its biology is constantly undergoing modification, and the therapeutic value of FGF21 is being hotly discussed.

(1)2000 Identification as a novel FGF, FGF-21, preferentially expressed in the liver.

(2)2005 FGF21 is a novel metabolic regulator of glucose homeostasis and insulin sensitivity.

(3)2006 FGF21 delays the initiation of hepatocarcinogenesis

(4)2007 β-Klotho functions as a cofactor essential for FGF21 activity.

(5, 6)2007 FGF21 is a "missing link" in the biology of fasting

(7)2008 Serum FGF21 levels are increased in type 2 diabetes mellitus

(8)2008 Serum FGF21 levels are increased in obesity and are independently associated with metabolic syndrome.

(9)2008 Systemic administration of FGF21 corrects obesity in mice

(10)2009 FGF21 protects pancreatic acini in acute pancreatitis.

(11)2010 FGF21 targets the central nervous system to realize the metabolic benefits

(12, 13)2010 Hepatic and serum FGF21 levels are increased in NAFLD

(14)2011 The circadian rhythm of FGF21 in humans

(15)2011 Serum FGF21 levels are increased in human mitochondrial disorders

(16, 17)2012 FGF21 is a key mediator of the physiologic and pharmacologic actions of PPARγ in both adipose tissue and bone.

(18)2012 FGF21 extends lifespan in mice

(19, 20)2012 FGF21-mimetic antibody and analogue are developed for treating diabetes and obesity

(21, 22)2013 adiponectin is implicated in FGF21 actions

(23)2013 protects against cardiac damage.

(24, 25)2013 FGF21 defines an liver-neuroendocrine axis in response to nutritional challenge

(26, 27)2015 FGF21 requires neither UCP1 nor brite adipocytes to elicit weight loss and improve glucose homeostasis.

(28, 29)2016 FGF21 regulates sweet and alcohol preference

(30, 31)2016 Fibroblast activation protein cleaves and inactivates human FGF21

(32)2016 Defining nutritional and metabolic context of FGF21

(33)2018 FGF21 governs water consumption in response to ketogenic diet and alcohol

(34)2018 FGF21 protects against hypertension

(35)2019 Pegbelfermin, a PEGylated human fibroblast growth factor 21 (FGF21) analogue, alleviates non-alcoholic steatohepatitis in phase 2a trial

(36)2020 FGF21 has therapeutic value for prevention and treatment of pancreatitis.

(37)2021 FGF21 drives cell competition

(38, 39)2021 Nanoparticle drug delivery system is applied in FGF21 treatment

References for Figure 1 (in chronological order).

1. Nishimura T, Nakatake Y, Konishi M, Itoh N. Identification of a novel FGF, FGF-21, preferentially expressed in the liver. Biochimica et Biophysica Acta (BBA) - Gene Structure and Expression2000. p. 203-6.
2. Kharitonenkov A, Shiyanova TL, Koester A, Ford AM, Micanovic R, Galbreath EJ*, et al.* FGF-21 as a novel metabolic regulator. The Journal of clinical investigation. 2005;115(6):1627-35.
3. Huang X, Yu C, Jin C, Yang C, Xie R, Cao D*, et al.* Forced expression of hepatocyte-specific fibroblast growth factor 21 delays initiation of chemically induced hepatocarcinogenesis. Mol Carcinog. 2006;45(12):934-42.
4. Ogawa Y, Kurosu H, Yamamoto M, Nandi A, Rosenblatt KP, Goetz R*, et al.* BetaKlotho is required for metabolic activity of fibroblast growth factor 21. Proc Natl Acad Sci U S A. 2007;104(18):7432-7.
5. Inagaki T, Dutchak P, Zhao G, Ding X, Gautron L, Parameswara V*, et al.* Endocrine Regulation of the Fasting Response by PPARα-Mediated Induction of Fibroblast Growth Factor 21. Cell metabolism. 2007;5(6):415-25.
6. Badman MK, Pissios P, Kennedy AR, Koukos G, Flier JS, Maratos-Flier E. Hepatic Fibroblast Growth Factor 21 Is Regulated by PPARα and Is a Key Mediator of Hepatic Lipid Metabolism in Ketotic States. Cell metabolism. 2007;5(6):426-37.
7. Chen WW, Li L, Yang GY, Li K, Qi XY, Zhu W*, et al.* Circulating FGF-21 levels in normal subjects and in newly diagnose patients with Type 2 diabetes mellitus. Exp Clin Endocrinol Diabetes. 2008;116(1):65-8.
8. Zhang X, Yeung DC, Karpisek M, Stejskal D, Zhou ZG, Liu F*, et al.* Serum FGF21 levels are increased in obesity and are independently associated with the metabolic syndrome in humans. Diabetes. 2008;57(5):1246-53.
9. Coskun T, Bina HA, Schneider MA, Dunbar JD, Hu CC, Chen Y*, et al.* Fibroblast Growth Factor 21 Corrects Obesity in Mice. Endocrinology. 2008;149(12):6018-27.
10. Johnson CL, Weston JY, Chadi SA, Fazio EN, Huff MW, Kharitonenkov A*, et al.* Fibroblast growth factor 21 reduces the severity of cerulein-induced pancreatitis in mice. Gastroenterology. 2009;137(5):1795-804.
11. Sarruf DA, Thaler JP, Morton GJ, German J, Fischer JD, Ogimoto K*, et al.* Fibroblast growth factor 21 action in the brain increases energy expenditure and insulin sensitivity in obese rats. Diabetes. 2010;59(7):1817-24.
12. Dushay J, Chui PC, Gopalakrishnan GS, Varela-Rey M, Crawley M, Fisher FM*, et al.* Increased fibroblast growth factor 21 in obesity

and nonalcoholic fatty liver disease. Gastroenterology. 2010;139(2):456-63.
13. Li H, Fang Q, Gao F, Fan J, Zhou J, Wang X, *et al.* Fibroblast growth factor 21 levels are increased in nonalcoholic fatty liver disease patients and are correlated with hepatic triglyceride. J Hepatol. 2010;53(5):934-40.
14. Yu H, Xia F, Lam KS, Wang Y, Bao Y, Zhang J, *et al.* Circadian rhythm of circulating fibroblast growth factor 21 is related to diurnal changes in fatty acids in humans. Clin Chem. 2011;57(5):691-700.
15. Suomalainen A, Elo JM, Pietiläinen KH, Hakonen AH, Sevastianova K, Korpela M, *et al.* FGF-21 as a biomarker for muscle-manifesting mitochondrial respiratory chain deficiencies: a diagnostic study. Lancet Neurol. 2011;10(9):806-18.
16. Dutchak PA, Katafuchi T, Bookout AL, Choi JH, Yu RT, Mangelsdorf DJ, *et al.* Fibroblast growth factor-21 regulates PPARγ activity and the antidiabetic actions of thiazolidinediones. Cell. 2012;148(3):556-67.
17. Wei W, Dutchak PA, Wang X, Ding X, Wang X, Bookout AL, *et al.* Fibroblast growth factor 21 promotes bone loss by potentiating the effects of peroxisome proliferator-activated receptor γ. Proc Natl Acad Sci U S A. 2012;109(8):3143-8.
18. Zhang Y, Xie Y, Berglund ED, Coate KC, He TT, Katafuchi T, *et al.* The starvation hormone, fibroblast growth factor-21, extends lifespan in mice. Elife. 2012;1:e00065.
19. Foltz IN, Hu S, King C, Wu X, Yang C, Wang W, *et al.* Treating diabetes and obesity with an FGF21-mimetic antibody activating the βKlotho/FGFR1c receptor complex. Sci Transl Med. 2012;4(162):162ra53.
20. Hecht R, Li YS, Sun J, Belouski E, Hall M, Hager T, *et al.* Rationale-Based Engineering of a Potent Long-Acting FGF21 Analog for the Treatment of Type 2 Diabetes. PLoS One. 2012;7(11):e49345.
21. Lin Z, Tian H, Lam Karen SL, Lin S, Hoo Ruby CL, Konishi M, *et al.* Adiponectin Mediates the Metabolic Effects of FGF21 on Glucose Homeostasis and Insulin Sensitivity in Mice. Cell metabolism. 2013;17(5):779-89.
22. Holland WL, Adams AC, Brozinick JT, Bui HH, Miyauchi Y, Kusminski CM, *et al.* An FGF21-adiponectin-ceramide axis controls energy expenditure and insulin action in mice. Cell metabolism. 2013;17(5):790-7.
23. Planavila A, Redondo I, Hondares E, Vinciguerra M, Munts C, Iglesias R, *et al.* Fibroblast growth factor 21 protects against cardiac hypertrophy in mice. Nature communications. 2013;4:2019.

24. Owen BM, Bookout AL, Ding X, Lin VY, Atkin SD, Gautron L, *et al.* FGF21 contributes to neuroendocrine control of female reproduction. Nature medicine. 2013;19(9):1153-6.
25. Bookout AL, de Groot MH, Owen BM, Lee S, Gautron L, Lawrence HL, *et al.* FGF21 regulates metabolism and circadian behavior by acting on the nervous system. Nature medicine. 2013;19(9):1147-52.
26. Véniant MM, Sivits G, Helmering J, Komorowski R, Lee J, Fan W, *et al.* Pharmacologic Effects of FGF21 Are Independent of the "Browning" of White Adipose Tissue. Cell metabolism. 2015;21(5):731-8.
27. Samms RJ, Smith DP, Cheng CC, Antonellis PP, Perfield JW, 2nd, Kharitonenkov A, *et al.* Discrete Aspects of FGF21 In Vivo Pharmacology Do Not Require UCP1. Cell Rep. 2015;11(7):991-9.
28. von Holstein-Rathlou S, BonDurant LD, Peltekian L, Naber MC, Yin TC, Claflin KE, *et al.* FGF21 Mediates Endocrine Control of Simple Sugar Intake and Sweet Taste Preference by the Liver. Cell metabolism. 2016;23(2):335-43.
29. Talukdar S, Owen BM, Song P, Hernandez G, Zhang Y, Zhou Y, *et al.* FGF21 Regulates Sweet and Alcohol Preference. Cell metabolism. 2016;23(2):344-9.
30. Dunshee DR, Bainbridge TW, Kljavin NM, Zavala-Solorio J, Schroeder AC, Chan R, *et al.* Fibroblast Activation Protein Cleaves and Inactivates Fibroblast Growth Factor 21. J Biol Chem. 2016;291(11):5986-96.
31. Coppage AL, Heard KR, DiMare MT, Liu Y, Wu W, Lai JH, *et al.* Human FGF-21 Is a Substrate of Fibroblast Activation Protein. PLoS One. 2016;11(3):e0151269.
32. Solon-Biet SM, Cogger VC, Pulpitel T, Heblinski M, Wahl D, McMahon AC, *et al.* Defining the Nutritional and Metabolic Context of FGF21 Using the Geometric Framework. Cell metabolism. 2016;24(4):555-65.
33. Song P, Zechner C, Hernandez G, Cánovas J, Xie Y, Sondhi V, *et al.* The Hormone FGF21 Stimulates Water Drinking in Response to Ketogenic Diet and Alcohol. Cell metabolism. 2018;27(6):1338-47.e4.
34. Pan X, Shao Y, Wu F, Wang Y, Xiong R, Zheng J, *et al.* FGF21 Prevents Angiotensin II-Induced Hypertension and Vascular Dysfunction by Activation of ACE2/Angiotensin-(1-7) Axis in Mice. Cell metabolism. 2018;27(6):1323-37.e5.
35. Sanyal A, Charles ED, Neuschwander-Tetri BA, Loomba R, Harrison SA, Abdelmalek MF, *et al.* Pegbelfermin (BMS-986036), a PEGylated fibroblast growth factor 21 analogue, in patients with non-alcoholic steatohepatitis: a randomised, double-blind, placebo-controlled, phase 2a trial. Lancet (London, England). 2019;392(10165):2705-17.

36. Hernandez G, Luo T, Javed TA, Wen L, Kalwat MA, Vale K*, et al.* Pancreatitis is an FGF21-deficient state that is corrected by replacement therapy. Sci Transl Med. 2020;12(525).
37. Ogawa M, Kawarazaki Y, Fujita Y, Naguro I, Ichijo H. FGF21 Induced by the ASK1-p38 Pathway Promotes Mechanical Cell Competition by Attracting Cells. Curr Biol. 2021;31(5):1048-57.e5.
38. Geng S, Qin L, He Y, Li X, Yang M, Li L*, et al.* Effective and safe delivery of GLP-1AR and FGF-21 plasmids using amino-functionalized dual-mesoporous silica nanoparticles in vitro and in vivo. Biomaterials. 2021;271:120763.
39. Gao J, Liu J, Meng Z, Li Y, Hong Y, Wang L*, et al.* Ultrasound-assisted C(3)F(8)-filled PLGA nanobubbles for enhanced FGF21 delivery and improved prophylactic treatment of diabetic cardiomyopathy. Acta Biomater. 2021;130:395-408.
